# Droplet Self-Propulsion on Slippery Liquid-Infused Surfaces with Dual-Lubricant Wedge-Shaped Wettability Patterns

**DOI:** 10.1021/acs.langmuir.3c02205

**Published:** 2023-10-24

**Authors:** Michele Pelizzari, Glen McHale, Steven Armstrong, Hongyu Zhao, Rodrigo Ledesma-Aguilar, Gary G. Wells, Halim Kusumaatmaja

**Affiliations:** † Institute for Multiscale Thermofluids, School of Engineering, The University of Edinburgh, Edinburgh EH9 3FB, U.K.; ‡ Department of Physics, Durham University, Durham DH1 3LE, U.K.

## Abstract

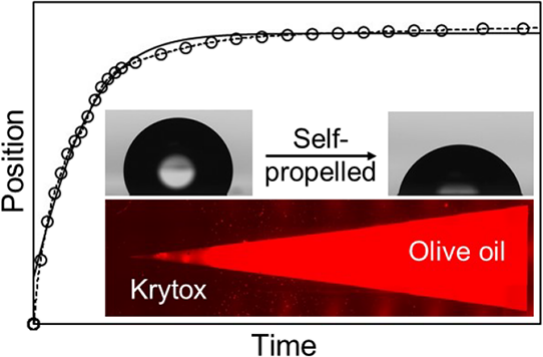

Young’s equation is fundamental to the concept of the wettability of a solid surface. It defines the contact angle for a droplet on a solid surface through a local equilibrium at the three-phase contact line. Recently, the concept of a liquid Young’s law contact angle has been developed to describe the wettability of slippery liquid-infused porous surfaces (SLIPS) by droplets of an immiscible liquid. In this work, we present a new method to fabricate biphilic SLIP surfaces and show how the wettability of the composite SLIPS can be exploited with a macroscopic wedge-shaped pattern of two distinct lubricant liquids. In particular, we report the development of composite liquid surfaces on silicon substrates based on lithographically patterning a Teflon AF1600 coating and a superhydrophobic coating (Glaco Mirror Coat Zero), where the latter selectively dewets from the former. This creates a patterned base surface with preferential wetting to matched liquids: the fluoropolymer PTFE with a perfluorinated oil Krytox and the hydrophobic silica-based GLACO with olive oil (or other mineral oils or silicone oil). This allows us to successively imbibe our patterned solid substrates with two distinct oils and produce a composite liquid lubricant surface with the oils segregated as thin films into separate domains defined by the patterning. We illustrate that macroscopic wedge-shaped patterned SLIP surfaces enable low-friction droplet self-propulsion. Finally, we formulate an analytical model that captures the dependence of the droplet motion as a function of the wettability of the two liquid lubricant domains and the opening angle of the wedge. This allows us to derive scaling relationships between various physical and geometrical parameters. This work introduces a new approach to creating patterned liquid lubricant surfaces, demonstrates long-distance droplet self-propulsion on such surfaces, and sheds light on the interactions between liquid droplets and liquid surfaces.

## Introduction

Friction is a significant source of energy dissipation in engineering applications.^1^ In the context of liquids, friction results from the interaction between a liquid and a solid surface due to, e.g., flow through piping, flow around a solid, and condensation or evaporation. These examples typically involve the wetting of solid surfaces and the motion of contact lines of liquid droplets or films and are extremely important in industrial applications such as printing^2^ and coating,^3^ heat exchange,^4^ and microfluidics.^5,6^ One of the key equations underpinning the wetting of a solid surface by a droplet is Young’s law,^7^ i.e.,
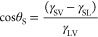
1where γ_IJ_ are the solid–liquid (SL), solid–vapor (SV), and liquid–vapor (LV) interfacial tensions. Equation 1 has a physical meaning only if the liquid does not completely wet the solid surface, or, equivalently, when the spreading coefficient for the liquid on the solid in the presence of a vapor, *S*_LS(V)_ = γ_SV_ – (γ_SL_ + γ_LV_), is less than zero.^8,9^ For partial and nonwetting surfaces, Young’s law introduces the concept of an equilibrium contact angle, θ_S_, for a droplet of a pure liquid on a flat, smooth, and homogeneous solid surface. Geometrically, the equilibrium contact angle is defined by the tangent to the liquid–vapor interface and the flat liquid–solid interface at the three-phase contact line.

In practice, however, a unique equilibrium contact angle, as defined by eq 1, is rarely observed. This is commonly attributed to the existence of heterogeneity on the solid surface arising from defects, contamination, roughness, or chemical heterogeneity. Such imperfections lead to the phenomenon of contact-line pinning, which results in a range of possible measured static contact angles bounded between the receding contact angle, θ_R_, and advancing contact angle, θ_A_, where the contact line overcomes the pinning force and either recedes or advances on the solid surface, respectively.^8−10^ The receding contact angle θ_R_ is defined as the last value measured immediately prior to the contact line advancing on the surface when volume is removed from a droplet infinitesimally slowly. Similarly, the advancing contact angle θ_A_ is defined as the last value measured immediately prior to the contact line advancing on the surface when the volume is added to a droplet infinitesimally slowly. The pinning of the contact line by the surface static friction can be quantified by the contact angle hysteresis, Δθ_CAH_ = θ_A_ – θ_R_.^11,12^ Given the importance of creating surfaces with low contact line pinning, Wong et al.^13^ proposed a new type of surface with almost no contact line pinning created by the introduction of a layer of a liquid lubricant infused into a solid porous coating that prevents a droplet from coming into direct contact with the underlying solid. Such slippery liquid-infused porous surfaces (SLIPS) are a specific type of a liquid-infused (lubricant-impregnated) surface and were also independently proposed by Lafuma and Quéré.^14^ The full set of possible morphologies for droplets on a lubricant-impregnated surface has been described by Smith et al.^15^ SLIPS have received significant attention due to their potential applications, showing low sliding angles,^13,15−18^ self-healing properties through capillary wicking upon damage and resistance to external pressure,^13^ and anti-icing^19^ and antibiofouling^20^ properties.

In a series of reports, some of the current authors have defined experimentally,^21^ and justified theoretically,^22,23^ an apparent contact angle on SLIPS and developed a coherent set of concepts on the wettability of surfaces of thin liquid films. This has included a liquid Young’s law,^22−24^ shaped-liquid surfaces whereby droplets self-propel on gradient SLIPS ridges,^25^ bidirectional self-propelled droplet motion toward areas of greater wettability on composite solid–liquid surfaces,^26^ and a critical surface tension determined by Zisman plots for SLIPS.^27^ These concepts complement work of other authors on the fundamentals of wettability of SLIPS, such as contact angles on SLIPS,^28^ slippery Wenzel,^29^ and slippery Cassie–Baxter^30^ surfaces. Of particular interest to the present article is prior literature work by Paulssen et al.^31^ on the patterning of liquid lubricants in a composite SLIP surface by patterning the underlying substrate with features of contrasting wettability to control the liquid–liquid displacement process spatially. In that work, a thin porous polymer layer was rendered superhydrophobic or hydrophilic through an esterification process and a UV-induced thiolyne reaction with a photomask. These surfaces were thereafter infused with pairs of different oils to provide spatially controlled SLIPS patterns and then used to create droplet microarrays. The same group also demonstrated droplet sorting and manipulation on a two-phase SLIPS patterned with macroscopic pathways using alkylated regions within a perfluorinated background, and infusion with perfluoropolyether (Krytox) and either mineral oil or silicone oil, respectively.^32^ In these two references, common enabling steps included an esterification process and click-chemistry, which, while effective, are techniques requiring significant chemistry expertise.

Separate to developments on SLIP surfaces, a common topic of interest for solid surfaces with geometric patterns of wettability has been droplet self-propulsion.^33,34^ However, given the limited range for wettability adjustment, a key challenge for self-propulsion has been to achieve long-distance transport and a limiting factor is then the static and kinetic friction forces acting against initiating and maintaining droplet motion.^32,35,36^ Therefore, the concept of using wettability contrasts on patterned composite SLIP surfaces is attractive for droplet self-propulsion due to the prospect of minimal static and kinetic friction due to droplet motion on lubricants rather than on solids. One of the simplest geometrical shapes to create a preferential motion for a droplet is a triangle. Previously, this has been considered in the context of both two-dimensional and three-dimensional surfaces, the latter especially in water harvesting applications.^37−39^ On a planar surface, the droplet motion is driven by a difference in wettability between the inside and outside areas of the triangle, a shape we refer to as a wedge-shaped pattern.^40−42^ If a droplet remains on a triangular pattern, which is more wettable than the background surface, there is a driving force to move a droplet from the apex (tip) of the triangle to the internal triangle region at the wider end of the triangle.^43−46^ However, motion occurs only when the force is sufficient to overcome droplet pinning.

In this work, we first consider the concept of wettability on SLIPS and compare it to the established concepts of wettability on solid surfaces, focusing on the droplet behavior on macro-patterns of wettability. We then introduce a new method of fabrication of patterned surfaces using a simple physical deposition and patterning of Teflon AF1600 followed by dip (or spray) coating of a commonly used commercial superhydrophobic particle coating (Glaco). We then convert these surfaces to patterned composite SLIP surfaces by the sequential infusion of pairs of oils, namely, Krytox and olive oil, which are the matching liquids to the Teflon AF1600 and Glaco solid substrate coatings, respectively. This approach provides an alternative to the esterification and click-chemistry approach to creating composite SLIPS and requires less sophisticated chemical expertise.^31,32^ We demonstrate that droplets on a macroscopically patterned composite SLIPS spontaneously move to the area of higher liquid wettability, as defined by the lowest value of contact angle in the liquid Young’s law. We then study the dynamics of a droplet on a composite lubricant wedge-shaped pattern, where the motion is driven by the gradient geometry and by the presence of two different lubricants inside and outside the wedge. On these wedge-shaped wettability patterned composite SLIPS, a droplet self-propels along the wettability gradient without significant pinning. Finally, we develop an analytical model for droplet self-propulsion that captures the time evolution of the droplet on the wedge-shaped pattern and we show that droplet position-time data are well-described by a simple tanh() law. This theoretical approach should also apply to self-propulsion on wedge-shaped wettability gradient on solid surfaces, although a minimum force pinning term and dissipation during the motion may need further consideration.

## Principles of Patterned Lubricant Surfaces

In this section, we recap ideas of a liquid Young’s law describing a contact angle for a droplet on a surface composed of a thin lubricant layer and explain how this allows the wettability of a SLIP surface to be defined. We then consider the criteria for the creation of a surface with two lubricants localized to different regions of the surface.

### A Single Infused Liquid

**Figure 1:**
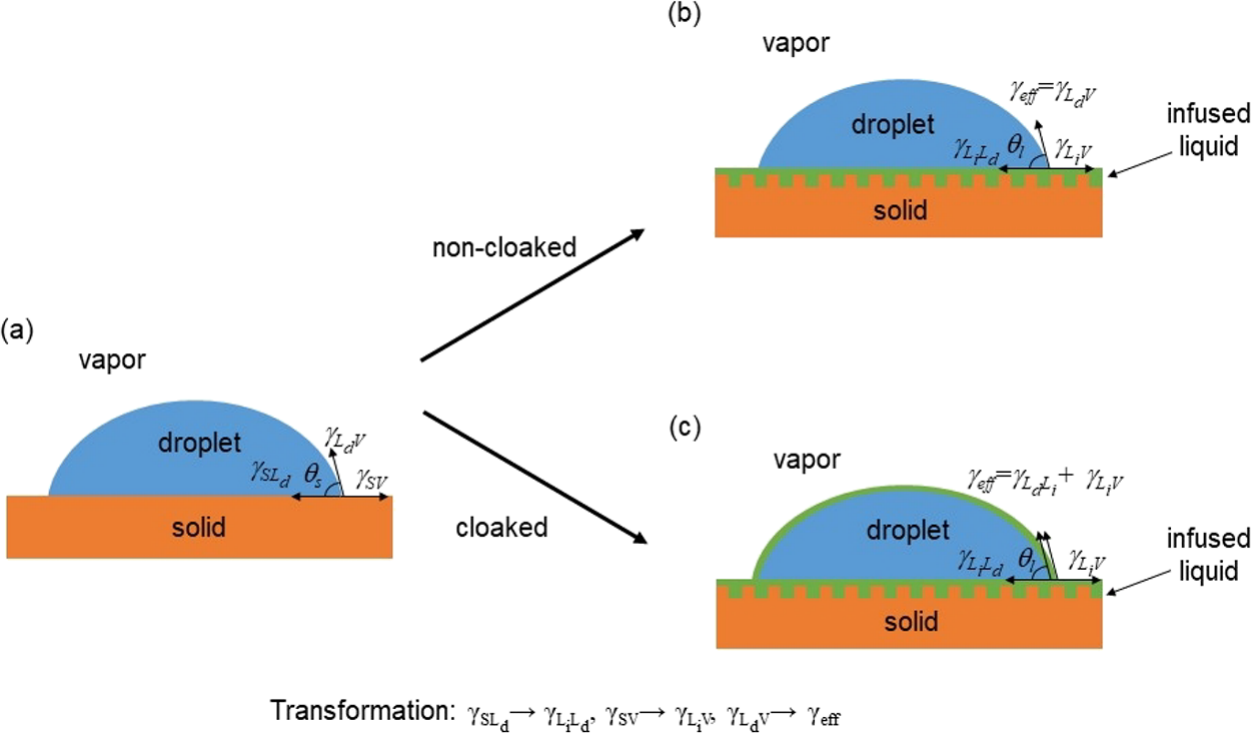
Young’s law and liquid Young’s law. Contact angles resulting from interfacial tensions acting on a droplet resting on (a) a solid surface, (b) a thin film of lubricant arising from an infused-liquid surface, which does not cloak the droplet, and (c) a thin film of lubricant arising from an infused-liquid surface, which does cloak a droplet.

On SLIPS with an infinitesimally thin film of the infused liquid coating the solid, the droplet liquid (*L*_d_) contact with a solid surface (*S*) is replaced by droplet contact with an infused liquid (*L*_i_), leading to the modified Young’s law (eq 1), or the liquid Young’s law,^22−24^ i.e.,
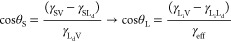
2where θ_S_ is the contact angle on the solid, θ_L_ is the contact angle on the infused liquid (Figure 1), and γ_eff_ is the effective liquid–vapor surface tension. Equation 2 converts Young’s law to a liquid Young’s law through both the symbolic substitution *S* → *L*_i_ and the replacement of the droplet liquid–vapor surface tension by an effective surface tension, i.e.,
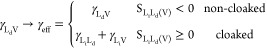
3

The need for γ_eff_ arises from the fact that a film of infused liquid will spread from the SLIP surface over the droplet–vapor interface when the spreading coefficient, *S*_L_i_L_d_(V)_ = γ_L_d_V_ – (γ_L_d_L_*i*__ + γ_L_i_V_) ≥ 0, a process referred to as cloaking the droplet. Experimentally, the measured contact angle for a droplet on a SLIP surface with a thin layer of the infused liquid is in excellent agreement with that predicted by the liquid Young’s law^23^ due to the extremely low pinning (and hence low contact angle hysteresis). This contrasts with the familiar situation of a smooth solid surface where significant pinning can occur, leading to measured static contact angles significantly different from those predicted by Young’s law. In the case of SLIP surfaces with thick films of the infused liquid, the liquid Young’s law gives an upper bound on the measured contact angle and measured angles are then lower and depend upon the thickness of the excess infused liquid.^22,47^

The concept of a contact angle on a SLIPS (or thin liquid film surface) enables the use of other concepts normally associated with the wetting of a solid surface provided that the droplet contacting the surface is immiscible with the infused liquid and does not displace the latter on the solid. For example, a droplet will self-propel along a gradient in the (liquid Young’s law) contact angle to regions of greater wettability until it finally comes to rest on a region of the surface where it is not subjected to the gradient anymore.^25,26^ Similarly, if a topographic surface structure, such as an array of ridges, is created using one length scale and a SLIPS coating is applied to it using a smaller-scale structure over the large length scale, droplets in both the suspended Cassie–Baxter^30^ state or the penetrated Wenzel^29^ state can be observed. The symbolic replacement, γ_SL_d__ → γ_L_i_L_d__, has also motivated the use of contact angle measurements on SLIPS as a method to determine the liquid–liquid interfacial tensions and to define a critical surface tension (minimal value of surface tension between a liquid and a solid for the liquid to wet the solid) on SLIPS.^27^

For a stable SLIP surface to be created with a single infused liquid, three design criteria were originally identified: (1) the lubricant (infused) liquid must wick into, wet, and stably adhere within the substrate, (2) the solid (i.e., substrate texture or porous coating) must be preferentially wetted by the lubricating (infused) liquid rather than by the (droplet) liquid one wants to repel, and (3) the lubricating (infused) and impinging test liquids (i.e., droplet liquids) must be immiscible.^13^ In stating these conditions, apart from the condition that the infused liquid is nonvolatile (at least on the time scale of interest), there are remarkably few limitations on the type of infused liquid that can be used as a lubricant. The selection of the lubricant can range from organic oils to synthetic oils, according to the requirements of each specific application.^48^

### Multiple Infused Liquids and Substrate Stability

Creating a stable composite SLIPS with two or more spatially localized infused-liquid lubricants requires more than the three design criteria applicable to a single infused-liquid SLIPS. The three original design criteria can be modified:

1. Each lubricating (infused) liquid must wick into, wet, and stably adhere within the substrate.
2. The solid (i.e., substrate texture or porous coating) must be preferentially wetted by the lubricating (infused) liquids rather than by the impinging (droplet) liquid one wants to repel.
3. Lubricating (infused) liquids and impinging test liquids (i.e., droplet liquids) must form an immiscible set of liquids.

However, in addition, to create SLIP surface patterns on a solid,

4. Each lubricant (infused) liquid must preferentially wet (compared to the other lubricants) a desired spatial region of the solid (i.e., substrate texture or porous coating) in air.

Design criteria (4) can be achieved by matching the surface chemistry of the spatial regions of the solid to that of the infused liquids. Thus, we expect a perfluorinated liquid lubricant, such as Krytox GPL103 (PFPE), to preferentially wet a fluoropolymer Teflon surface compared to silicone oils (CH_3_ terminal groups) and mineral oils (alkane and cycloalkane based). Similarly, we expect silicone and mineral oils to preferentially wet a surface constituted by methyl-terminated hydrophobic silica nanoparticles compared to Krytox. Design criteria (1), (2), and (4) could be summarized based on the interfacial tensions and spreading coefficients.

In addition to these four design criteria, we envisage that for the composite SLIPS to be absolutely stable when in contact with a liquid of interest, there need to be two further design criteria:

5. Each lubricant (infused) liquid must retain its preferential wetting of the desired spatial region of the solid (i.e., substrate texture or porous coating) when immersed in the impinging test liquids (i.e., droplet liquid).
6. The impinging test liquids (i.e., droplet liquids) should preferentially wet each lubricant (compared to intercalating a layer of one of the other lubricants between the localized lubricant and the test liquid).

These two further design criteria ((5) and (6)) foreshadow the possibility that the infused liquids in a composite SLIPS, which is stable in air, might rearrange in a variety of complex ways when immersed under a droplet of a given liquid. This rearrangement might involve the displacement of an infused liquid from what was its previous preferred spatial location by another infused liquid, or it might be the formation of two (or more layers) of infused liquids between a spatial region of the substrate and the droplet liquid. However, working with all six design criteria introduces a complex set of material constraints. Hence, in practice, when designing our experiments, *a priori* we focus on criteria (1)–(4). We then seek to understand how the resulting surfaces behave experimentally and whether they meet criteria (5) and (6) for stability.

### Multiple Infused Liquids and Cloaking

**Figure 2:**
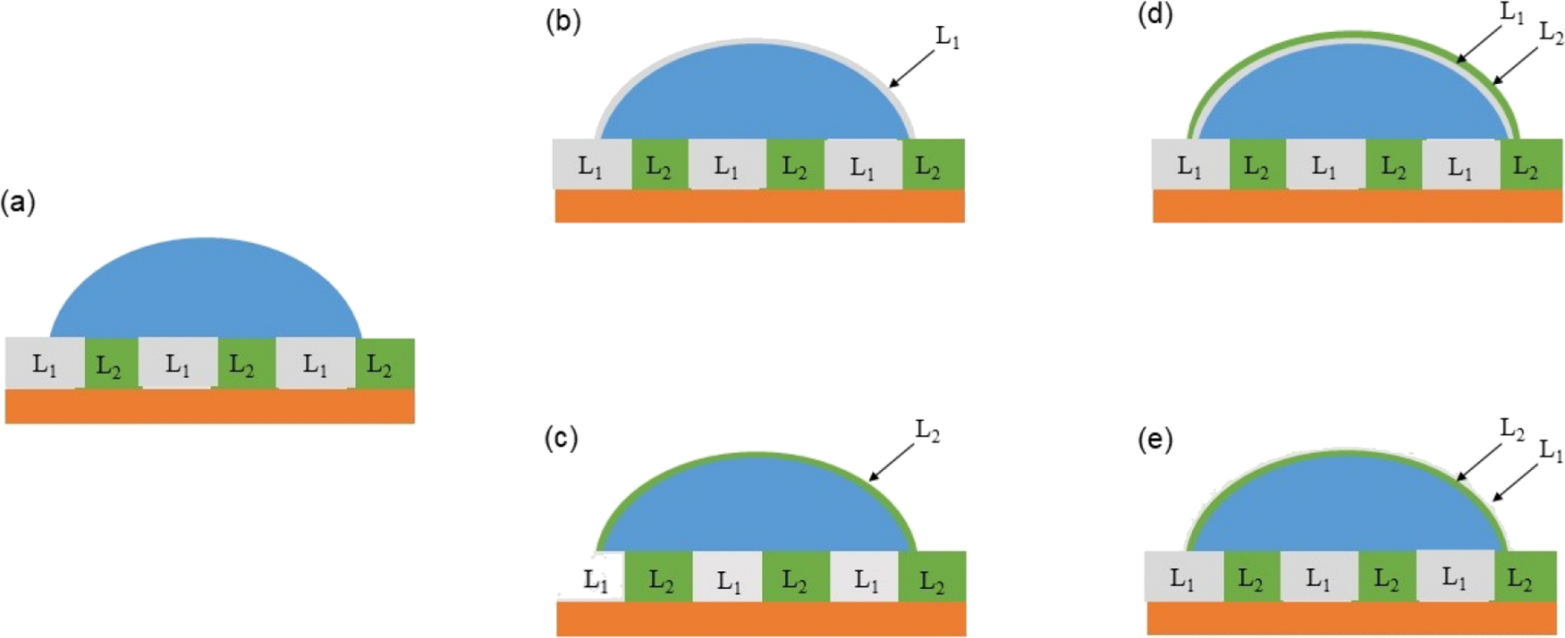
Five cloaking possibilities for a droplet on a composite SLIPS. (a) Non-cloaked. (b) Cloaked by infused-liquid 1. (c) Cloaked by infused-liquid 2. (d) Cloaked by infused-liquid 1 and then by infused-liquid 2. (e) Cloaked by infused-liquid 2 and then by infused-liquid 1. The droplet encapsulations in (b) and (c) are analogous to single interface core–shell compound drops, and those in (d) and (e) are analogous to double-interface core–shell compound drops.

A complication from the presence of more than one infused liquid is that more than one type of cloaking of the droplet liquid–vapor interface may become possible depending on the various interfacial tensions. In the case of a composite SLIPS using two infused liquids, we envisage five cloaking possibilities (Figure 2): (1) uncloaked, (2) cloaked with infused-liquid 1, (3) cloaked with infused-liquid 2, (4) cloaked with infused-liquid 1 and then with infused-liquid 2, and (5) cloaked with infused-liquid 2 and then with infused-liquid 1. States (2)–(5) are analogous to the concept of single- and double-interface core–shell compound drops,^49^ although the encapsulation involves a thin film of the infusing liquid(s). Concepts from fluid–fluid interfaces in emulsions are also relevant (in a similar manner as the analogy between liquid marbles and Pickering emulsions), although here the problem involves encapsulated single droplets.^50,51^

Thus, using the convention for the spreading coefficient for fluid “a” on fluid (or solid) “b” in the presence of fluid “c” of *S*_ab(c)_ = γ_bc_ – (γ_ba_ + γ_ac_) where γ_ij_ are the interfacial tensions, the effective droplet-vapor surface tension may take one of five values, as shown in Table 1. The equilibrium cloaking state will be the one with the lowest surface energy per unit area for γ_eff_ although these considerations do not address the kinetics of the process.

**Table 1:** Possible Values of the Effective Droplet Surface Tension, γ_eff_, Based on Cloaking Conditions

γ_eff_ definition	spreading coefficients	cloaking condition
γ_L_d_V_	*S*_L_1_L_d_(V)_ < 0, *S*_L_2_L_d_(V)_ < 0	non-cloaked
γ_L_1_L_d__ + γ_L_1_V_	*S*_L_1_L_d_(V)_ > 0, *S*_L_1_L_d_(V)_ > *S*_L_2_L_d_(V)_, *S*_L_1_L_2_(V)_ < 0	single-cloaked by liquid 1
γ_L_2_L_d__ + γ_L_2_V_	*S*_L_2_L_d_(V)_ > 0, *S*_L_2_L_d_(V)_ > *S*_L_1_L_d_(V)_, *S*_L_1_L_2_(V)_ < 0	single-cloaked by liquid 2
γ_L_1_L_d__ + γ_L_1_L_2__ + γ_L_2_V_	*S*_L_1_L_d_(V)_ + *S*_L_2_L_1_(V)_ > 0, *S*_L_d_L_1_(V)_ > *S*_L_d_L_2_(V)_, *S*_L_2_L_1_(V)_ > 0, *S*_L_1_L_2_(L_d_)_ > 0	double-cloaked by liquid 1 followed by liquid 2
γ_L_2_L_d__ + γ_L_1_L_2__ + γ_L_1_V_	*S*_L_2_L_d_(V)_ + *S*_L_1_L_2_(V)_ > 0, *S*_L_d_L_2_(V)_ > *S*_L_d_L_1_(V)_, *S*_L_1_L_2_(V)_ > 0, *S*_L_2_L_1_(L_d_)_ > 0	double-cloaked by liquid 2 followed by liquid 1

In the next section, we show how the general principles of composite SLIP surfaces with two lubricants can be implemented using a new materials method based upon lithographic patterning techniques and the use of a dewetting concept and the preferential partitioning of lubricants to different surface regions. We also implement two types of patterned surfaces whose (lubricant) wettability contrast and gradients are designed to induce the motion and self-propulsion of water droplets.

## Experimental Methods and Materials

### Fabrication of Composite SLIPS Using Dewetting

**Figure 3:**
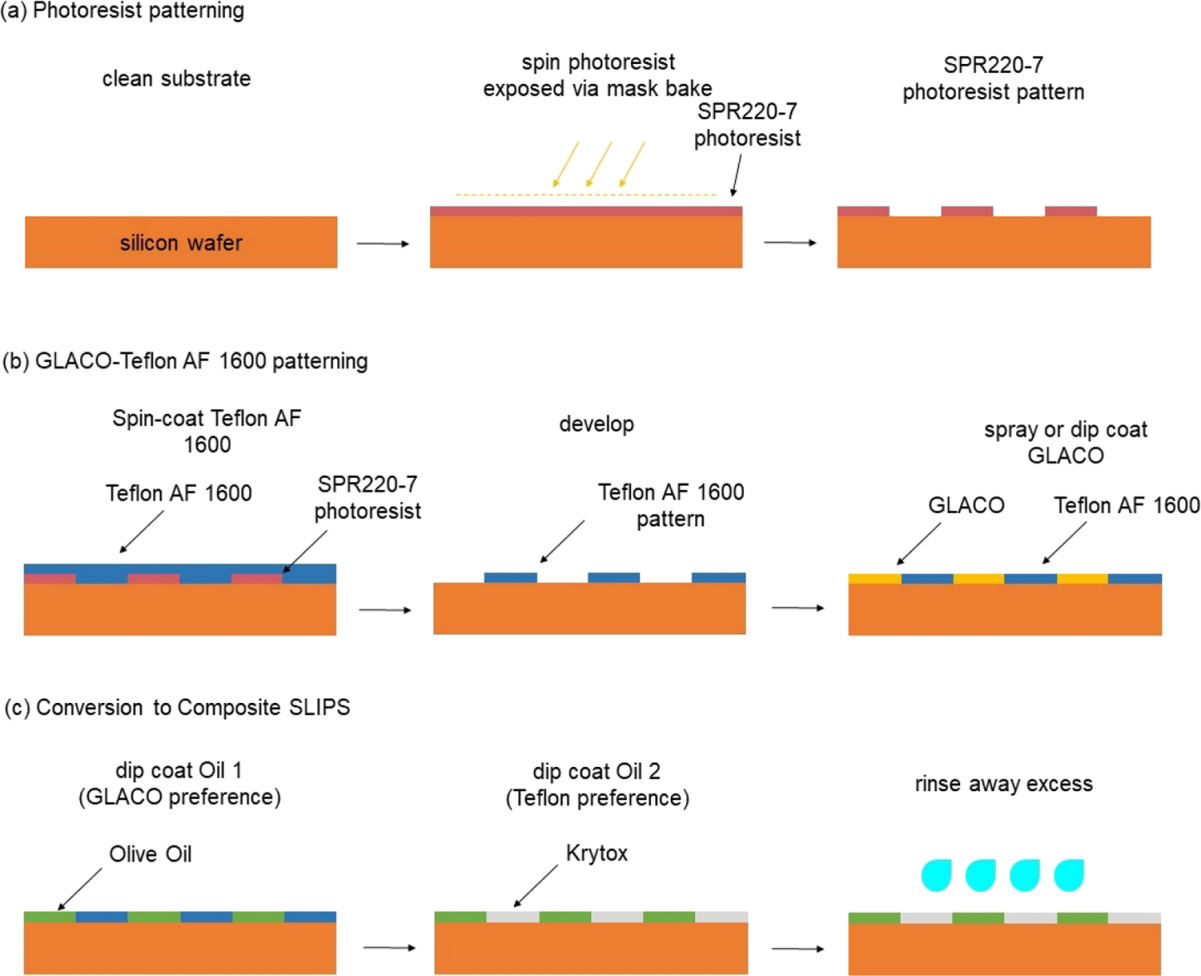
Schematic of the Glaco–Teflon AF1600-based SLIPS using dewetting and liquid–liquid displacement. (a) Photolithographic creation of the patterned Teflon AF1600 surface. (b) Dewetting to create a patterned Glaco–Teflon AF1600 surface. (c) Conversion to composite SLIPS by infusing a liquid (e.g., silicone oil, olive oil, sunflower oil), which preferentially wets the superhydrophobic Glaco, and then by infusing another liquid, which preferentially wets Teflon AF1600 and displaces the first oil from the Teflon AF1600 (e.g., Krytox)

Our concept for making patterned composite SLIPS is based on Teflon AF1600 prepared by mixing poly[4,5-difluoro-2,2-bis(trifluoromethyl)-1,3-dioxole-*co*-tetrafluoroethylene] with its solvent octadecafluorodecahydronaphthalene and Glaco Mirror Coat Zero (SOFT 99 Corp), a liquid solution of isopropyl alcohol (IPA), and hydrophobic silica nanoparticles. From our previous work, we have reported the ability of Glaco and Teflon AF1600 deposited on glass to preferentially stabilize different types of oils.^27^ Our composite SLIPS concept, shown in Figure 3, is as follows: (a) We use photolithography to create micro- or macro-patterns of SPR220-7 photoresist on glass or silicon substrates. (b) We spin-coat Teflon AF1600 onto the patterned surface and remove the photoresist to leave only the AF1600 pattern on the substrates. We then dip- or spray-coat the substrates with Glaco, where the solvent IPA dewets from the surface of Teflon AF1600 and leaves the Glaco coating only on the areas not coated with AF1600. (c) We dip-coat a first oil that is preferentially stabilized by Glaco at a withdrawal speed of 0.01 mm/s using a Dip Coater (L2006A1-UK, Ossila Ltd., UK) and then dip-coat with a second oil that is preferentially stabilized by Teflon AF1600 at the same withdrawal speed. Choosing appropriate lubricant oils as the infusing liquid that stabilize on either Teflon AF1600- or Glaco-patterned region allows the oil to displace the less preferential oil from the solid surface for which it has higher affinity.

### Preparation of Teflon AF1600/Glaco-Patterned Substrates

In a clean room environment, the photoresist (MEGAPOSIT SPR220-7, Kayaku Advanced Materials) is spin-coated on a 3 in. silicon wafer at 350 rpm for 120s with an acceleration of 100 rpm/s and then at 1000 rpm for 20 s with an acceleration of 100 rpm/s. After spin-coating, the substrate is then soft baked for 90 s at 115 °C. The photoresist-coated wafer is then exposed to UV patterns in a direct-write photolithography machine (MicroWriter ML3 Pro, Durham Magneto Optics Ltd.) according to a predesigned digital pattern at the energy level of between 350 and 700 mJ/cm^2^. The exposed photoresist-coated wafer is then developed using MF-26A (Kayaku Advanced Materials) for 60 s, and then rinsed with deionized water and blow-dried with compressed nitrogen. The thickness of photoresist is measured as 5.69 ± 0.03 μm using a stylus profilometer (DektakXT, Bruker).

The deposition of Teflon AF1600 is well established^52,53^ and performed before the GLACO deposition. The liquid Teflon is prepared with 0.5 wt % of poly[4,5-difluoro-2,2-bis(trifluoromethyl)-1,3-dioxole-*co*-tetrafluoroethylene] in octadecafluorodecahydronaphthalene. The solution is left overnight on a hot plate at 60 °C under continuous stirring before spin-coating onto the sample containing the photolithographic pattern.^52,54^ The liquid Teflon is spin-coated at 500 rpm for 10 s and then ramped up to 2000 rpm for 1 min. After the spin-coating process, the sample is dried in ambient conditions for approximately 5 min in a fume hood and then placed on a hot plate at 155 °C for 20 min to completely cure the Teflon.^53^ The remaining patterned photoresist is then removed by placing the sample in a glass Petri dish in an acetone bath for 5 min, with the Petri dish manually agitated. The sample is then rinsed gently with clean acetone for 20 s to remove any remaining traces of photoresist. Finally, the sample is rinsed gently with deionized (DI) water to remove any contaminants from the sample. The measured advancing and receding contact angles on the Teflon AF1600 are 131.5° ± 1.0° and 106.9° ± 1.7°, respectively, with no systematic differences for coatings prepared with spin speeds ranging from 500 to 5000 rpm.

To achieve greater control of the thickness and wettability properties of the Glaco layer, we tested both spray-coating^55−58^ and dip-coating^59^ approaches to depositing GLACO on glass and silicon substrates without any patterning. Both the spray coating and dip coating methods resulted in adequate deposition of GLACO and created a superhydrophobic surface coating on glass slides and silicon wafers. The spray coating method provided superhydrophobic coatings with static contact angles of (168.0° ± 1.3°) and negligible drop pinning, consistent with previous reports.^27,55−58^ However, the dip coating method provides the ability to choose the withdrawal speed, which controls the thickness of the liquid layer via the Landau–Levich–Derjaguin (LLD) equation,^23^ and the number of repeats of the dipping/withdrawal process allows more control over the GLACO layer thickness. A wide range of withdrawal velocities, *U*_W_, were selected from *U*_W_ = 0.01 to *U*_W_ = 5.00 mm/s. For the samples presented in this paper, a single dip-coating process in Glaco has been used with *U*_W_ = 1.00 mm/s. The dewetting of Glaco solutions from Teflon AF1600 is illustrated by the scanning electron microscope (SEM) image in the Supporting Information (Figure S1).

### Selection of Lubricant Oils as Infused Liquids

We selected the low surface tension perfluorinated oil Krytox,^13,48^ as the infused-liquid for Teflon AF1600 because of the presence of fluorine groups and its stability against displacement by alkanes.^27^ We measured the apparent contact angle for deionized water droplets on a Krytox/Teflon AF1600-based SLIP surface (herein denoted KT-SLIPS) at 119.2° ± 0.9°, consistent with a previous report,^27^ when the lubricant is thin and not in excess. The oils considered as possibilities to be the infused liquid for GLACO were an expanded set informed by our previous work on SLIPS.^26,27^ After testing a set of oils (silicone oil, olive oil, and sunflower oil), which all formed suitable stable coatings compatible with producing a composite SLIP surface, we focused our work on olive oil. The choice has been justified by the fact that the apparent contact angle of DI water droplets on olive oil GLACO-based SLIPS (herein denoted as OOG-SLIPS) was 83.9° ± 1.0° when the layer of oil was not in excess (i.e., minimum thickness oil) and so gave a reasonable contact angle contrast to that on Krytox. Values of theoretical and measured apparent contact angles and data for interfacial tensions are reported in Tables 2 and 3. The interfacial tension values were measured with the pendant drop method using a KRÜSS DSA 25. Both Krytox and olive oil show a value of spreading coefficient *S*_OW(V)_ higher than zero, which means that water droplets are cloaked with both Krytox and olive oil. The theoretical values of apparent contact angles θ_th_ calculated using the liquid Young’s law (eq 2) are in good agreement with the experimentally measured angles θ_exp_. The value of interfacial tension between Krytox and olive oil is γ_KO_ = 12.62 ± 0.01 mN/m. By selecting olive oil to be complementary to Krytox, we obtained the largest difference in contact angle from the oils considered for the two component SLIP surfaces in our composite SLIP surfaces. This allowed a large window in apparent contact angles and thus the largest difference in wettability. To ensure the two oils have appropriate preferential wetting properties on the substrate materials, we confirmed that droplets of olive oil do not displace Krytox when deposited on Krytox-infused Teflon AF1600-based SLIPS and that droplets of Krytox do not displace olive oil when deposited on olive oil-infused GLACO-based SLIPS. Example side-profile images showing contact angles of various droplets on different surfaces supporting this conclusion are given in the Supporting Information (Figure S2).

**Table 2:** Theoretical and Measured Contact Angles of Water Droplets on Oils

oil	γ_OV_ (mN/m)	γ_WV_ (mN/m)	γ_WO_ (mN/m)	*S*_OW(V)_ (mN/m)	γ_eff_ (mN/m)	θ_th_ (°)	θ_exp_ (°)
Krytox	17.41 ± 0.02	72.7 ± 0.1	51.3 ± 0.1	4.00	68.7 ± 0.1	119.6 ± 0.1	119.2 ± 0.9
olive	32.19 ± 0.02	72.7 ± 0.1	21.8 ± 0.5	18.73	54.0 ± 0.5	78.9 ± 0.5	83.9 ± 1.0
silicone 20 cSt	20.22 ± 0.05	72.7 ± 0.1	38.1 ± 0.7	14.40	58.3 ± 0.7	107.9 ± 0.3	108.1 ± 0.9
sunflower	31.87 ± 0.03	72.7 ± 0.1	21.6 ± 0.1	19.25	53.5 ± 0.1	78.9 ± 0.1	83.6 ± 1.5

**Table 3:** Values of Interfacial Tensions between Pairs of Oils

oil 1	oil 2	γ_O1O2_ (mN/m)
Krytox	olive	12.62 ± 0.01
Krytox	silicone 20 cSt	7.13 ± 0.01
Krytox	sunflower	12.52 ± 0.01
silicone 20 cSt	olive	2.19 ± 0.01
silicone 20 cSt	sunflower	1.56 ± 0.01

### Simple Boundary and Wedge-Shaped Wettability Patterns

**Figure 4:**
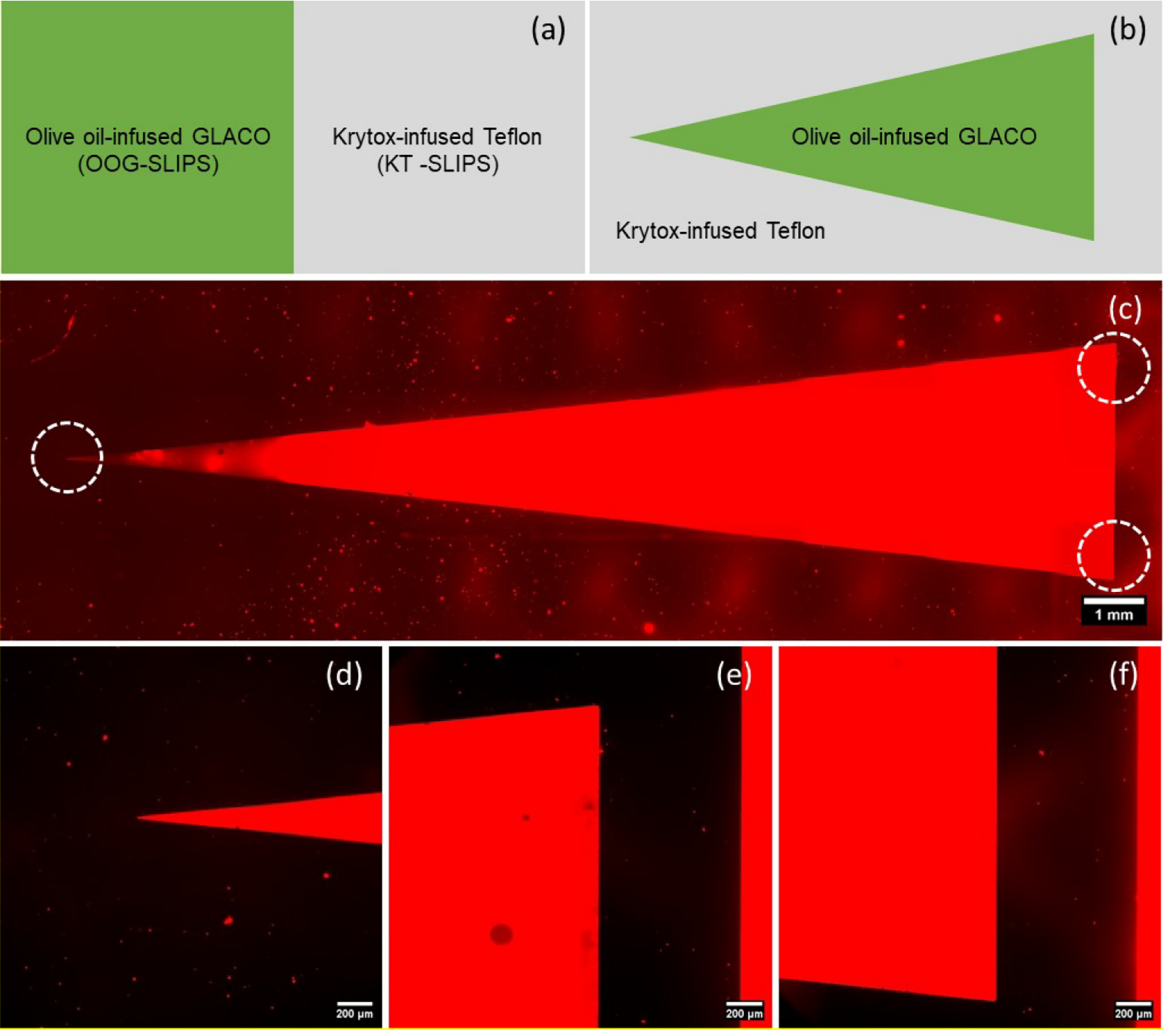
Schematic macro-patterns for composite SLIPS showing the (a) step boundary and (b) wedge of lower wettability. Fluorescence images of a wedge-shaped pattern with the olive oil mixed with Nile red dye: (c) composite image showing a full wedge, (d) an image of the apex region, and (e) and (f) image of the (step) boundaries between oils at the wider end of the wedge. In the fluorescence images, red shows the olive oil-coated regions and black shows the Krytox-coated regions.

To exemplify the effect of the difference in wettability on a patterned olive oil–Krytox Composite SLIPS (from now on called OOG−KT Composite SLIPS) on droplet equilibrium states and motion, two different geometries were tested. For the first case, we created a simple binary pattern with a composite SLIPS with a left-hand side composed of OOG-SLIPS and a right-hand side composed of KT-SLIPS (Figure 4a). This simple step change at the spatial boundary between lubricants should create a preferential wettability on the lower (liquid) contact angle olive oil surface. When a 4 μL DI water droplet was deposited on the boundary region between the two oils, we observed that it moved across to sit on the OOG-SLIPS region. The movement of the DI water droplet is immediate upon deposition (Supplementary Video 1 and Supporting Information Figure S3). For the second case, we created 11 wedge patterns with olive oil within the wedge and Krytox outside and with wedge opening angles at the apex from 8 to 28° in steps of 2° (Figure 4b). For droplets larger than the wedge width, this geometry creates a wettability gradient driving a preferential self-propelled motion of water droplets along the wedge axis from the apex to the wider side of the wedge, with the more wettable OOG regions. Moreover, due to the symmetry of the system and the less wettable region external to the wedge region, a water droplet also self-centers itself with respect to the wedge axis. To confirm the two oils are patterned as desired, we prepared wedges using olive oil mixed with the fluorescent dye Nile red and performed fluorescence imaging using a Leica DMi8 microscope equipped with a Rhodamine cube (excitation wavelength of 488 nm and emission wavelength at 525 nm). Figure 4c shows an example fluorescence image of a wedge pattern (the apex has an opening angle of 12°) composed of a collection of multiple single images to achieve a sufficient field of view from the wide end of the wedge to its apex. The stitching process produces some minor artifacts visible in the otherwise black Krytox-infused region, and the exposure time of the image of the edges of the wedge and the apex is not optimized in this view. Figure 4d shows a single image of the apex region of the wedge with the exposure time optimized. Similarly, Figure 4e,f shows single images demonstrating that the (step) boundaries between the olive oil and Krytox regions are well-defined.

### Measurements of Drop Motion on Wedge-Shaped Patterns

For each experiment, a 4 μL ultrapure deionized water droplet was deposited at the apex of a wedge-shaped pattern using an ExiGo syringe pump (Cellix) with a Hamilton glass syringe of 500 μL. Droplet motion was simultaneously video-recorded from both a side-profile view and a top view using two Raspberry pi high-quality cameras. The position-time data for the droplet center was determined from the side-profile view video recorded at 60 frames per second over at least 1 min. For each video sequence, every frame was analyzed using the open-source software pyDSA^60^ (ellipse fitting method). Experiments were repeated three times for each wedge shape.

## Theory of Droplet Self-Propulsion on a Wedge-Shaped Pattern of Wettability

### Model Assumptions

**Figure 5:**
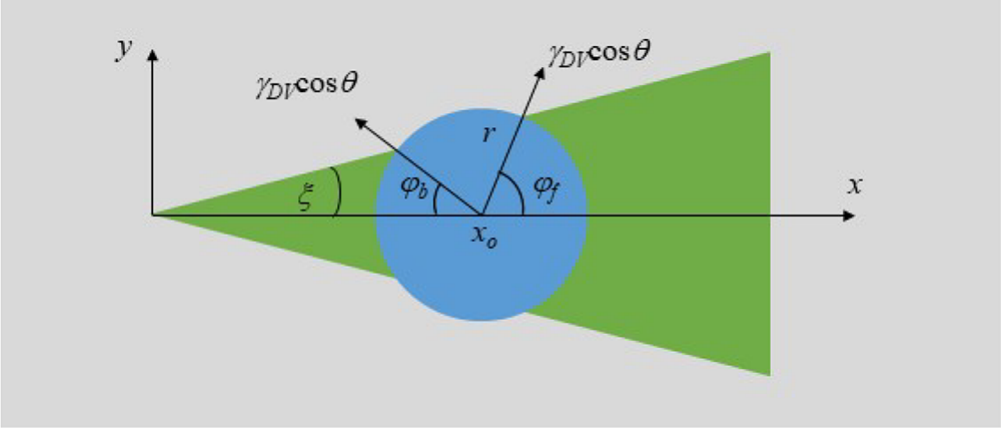
Schematic representation of the wedge geometry considering a circular droplet footprint. *x*_o_ is the geometrical center of the droplet that moves along the *x*-axis. ξ is the half angle at the apex of the wedge, and φ_f_ and φ_b_ are the angles at the front and back of the droplet in the *x*–*y* plane defined by the intersections between the droplet footprint and the wedge geometry. γ_DV_cos θ is the projection of the capillary force into the *x*–*y* plane.

We consider a small droplet spanning the full width of the wedge and define the *x*-axis from the apex toward the wider end of the wedge (Figure 5). In this case, the droplet will have a larger fraction of its contact line on the more wettable wedge region at its front than the fraction of its contact line on the more wettable wedge region at its back. The droplet will therefore have a driving force toward the positive *x* direction. To describe the droplet motion in time, we assume that (1) there is no evaporation or other loss of mass, (2) motion occurs slowly so that inertial and acceleration effects can be neglected, (3) motion is driven by the difference in contact angles (which in turn set up a Laplace pressure gradient) between the front and back of the droplet, (4) contact angles are in a quasi-equilibrium state at each moment in time, (5) the droplet retains a spherical cap shape with a circular contact area, (6) there is no change in lubricant state below the droplet, (7) there is no change in cloaking state on the droplet, and (8) sliding friction is negligible. For motion on the wedge, assumption (5) is unlikely to be accurate as we can anticipate that the droplet will elongate along the axis of the wedge and the contact line will be distorted by the edges of the wedge and tend to align to them particularly as the droplet comes to rest entirely on the inner region of the wedge. For a composite SLIPS-based wedge, assumption (6) depends on whether droplet motion causes any depletion or rearrangement of lubricants. Prior experience with SLIPS suggests depletion will occur to a limited extent. In the case of a composite SLIPS wedge, we expect assumptions (7) and (8) to be reasonably valid. We further discuss the validity of the applicability of the theory to the experimental data in the Discussion section with a particular emphasis on assumptions (5) and (6).

### Equation of Motion

To derive an equation of motion for a droplet on a wedge–shaped region of contrasting wettability (Figure 5), we write the conservation of momentum for the droplet mass as
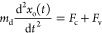
4where *m*_d_ is the mass of the droplet, *x*_o_(*t*) is the center position of the droplet in time, *F*_c_ is the capillary force due to contact angle differences, and *F*_v_ is the viscous friction arising from the drop on both lubricant components. Setting the acceleration term to zero, this equation reduces to
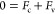
5The viscous friction force will be related to the speed of motion of the droplet and the viscosity of the droplet compared to the lubricants. Following the argument by Bjelobrk et al.,^61^ there are contributions from the droplet, the lubricant underneath the droplet, and the lubricant meniscus. The first two contributions lead to terms proportional to the droplet viscosity, while the final contribution gives rise to a term proportional to the lubricant viscosity. Given we have two different lubricants, we write the viscous force as
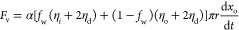
6where the subscripts i, o, and d represent the oil inside the wedge (olive oil), the background oil outside the wedge (Krytox), and the droplet (water), respectively; the η*’*s are the viscosities of the liquids (droplet and lubricants), *r* is the base radius of the droplet, and α is a numerical coefficient, which is expected to be ∼22–27.^61^ In principle, there are several possible choices that could be made for the definition of *f*_w_(*x*_o_). Here, we define it as the fraction of the lubricant meniscus inside the wedge, rather than a Cassie area weighted average, to maintain consistency with understanding of dissipation in SLIPS being dominantly due to the lubricant meniscus. The dissipation will depend on the ratio of lubricant viscosity to droplet viscosity and size of the wetting ridge^62^ and also whether the interface between the droplet and lubricant is rigidified by, for example, contaminants or surfactants, or allows momentum transfer across it. Equation 6 can be written as
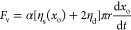
7where η_s_(*x*_o_) = *f*_w_η_i_ + (1 – *f*_w_)η_o_ is the average viscosity in the lubricant meniscus when the droplet is at position *x*_o_ along the wedge.

We now turn our attention to the driving capillary force. Integrating the component in the direction of motion of the effective droplet-vapor surface tension, γ_DV_ (see Table 1 with γ_eff_ = γ_DV_) around the base of the droplet gives
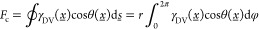
8where *x* is the (*x*,*y*) location on the surface and d*s* is on the droplet perimeter on the surface. For the wedge shape, the capillary force, eq 8, evaluates to (Supporting Information, Section 4)
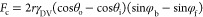
9

The azimuthal angles φ_b_ and φ_f_ depend on the wedge half-opening angle, ξ, the drop position *x*_o_*,* and base radius *r*, and so eq 9 can be rewritten as (Supporting Information)
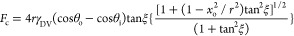
10Using eqs 7 and 10, the equation of motion, eq 5, then becomes
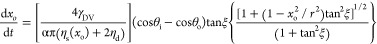
11Equation 11 has a directional term given by the difference in contact angles between the inside and outside of the wedge (for a discussion of bidirectional self-propulsion of droplets, see Sadullah et al.^26^).

### Analytical Solution for Small Wedge Opening Angles

To simplify eq 11, we assume that the wedge half-opening angle ξ is small compared to the characteristic size of the droplet and expand the square root and tan ξ terms assuming ξ*x*_o_/*r* ≪ 1 (Supporting Information),
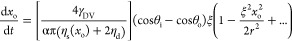
12To solve eq 12 analytically, we make two further assumptions. The first is that the (in principle, position-dependent) lubricant meniscus viscosity η_s_(*x*_o_) can be replaced by an average value < η_s_ > along the droplet trajectory; the second is that the droplet base radius, *r*, is approximately constant. This means there is a characteristic speed for droplet motion given by the ratio of the droplet effective surface tension to the effective viscosity, i.e., v* = γ_DV_/(<η_s_> + 2η_d_), which is dependent on both the lubricants and the droplet viscosities. It also means that to first order, corresponding to droplets close to the apex of the wedge, the (initial) speed scales with the wedge (half) opening angle, ξ. The solution to eq 12 with these assumptions is
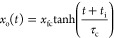
13where the final limiting position, *x*_fc_, is given by
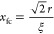
14and the time constant, τ_c_, is
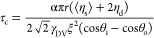
15Here, *t*_i_ is a constant of integration, which is zero if *x*_o_(0) = 0, but experimentally is determined from the fit to the measured data series. The limiting cases for eq 13 at early and late times (assuming *t*_i_ = 0) are
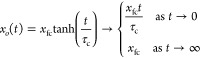
16Hence, the initial speed is *v*_*i*_ = *x*_fc_/τ_c_ and then the speed drops to zero with a time constant, τ_c_, as the droplet approaches its final position *x*_fc_. It is also interesting to note that the speed of droplet motion, *v*_o_, is given by the first derivative of eq 16
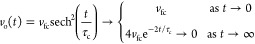
17

### Ansatz Solution for Non-Circular Contact Areas

Experimentally, we expect the fifth assumption that the droplet retains a spherical cap shape with a circular contact area to be broken. We can anticipate that for small droplets, the side-profile view will remain well-approximated by a circular arc, but from a top view, the contact area will be elongated along the wedge in the direction of motion. We therefore consider the consequences of using an elliptical contact area rather than a circular contact area. One important change is that the intersection between an ellipse and the wedge results in the replacement (Supporting Information),
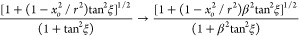
18where β = *r*/*b* and 2*r* is the major axis width (i.e., the diameter viewed in side profile across the wedge) and 2*b* is the minor axis width (i.e., diameter viewed along the wedge). We also change the radius parameter, *r*, in eq 7 and 9 to be an effective radii resulting in an overall scaling factor, which we define as λ. The equation of motion, eq 12, is therefore modified to
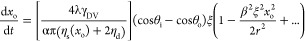
19Despite these modifications, the form of the equation of motion remains similar to the circular contact area case and means we obtain a solution
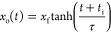
20where the final limiting position, *x*_f_, is given by
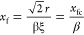
21and the time constant, τ, is
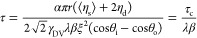
22In the above, it has been assumed that a top view gives an elliptical shape; however, we can also anticipate that the ellipse will be distorted with a wider front than rear. Thus, an ellipse based on the front of the droplet will have a larger minor axis width than an ellipse based on the back of the droplet. However, for the form of the tanh() solution, the important part in the argument above is the intersection between the ellipse and the wedge. A distorted ellipse will therefore lead to different β parameters for the front and rear of the droplet and, hence, an average β parameter for the droplet overall. Moreover, given sufficient wettability contrast, the distorted ellipse shape will be predominantly located within the wedge. This provides further support for the assumption that the effective lubricant meniscus viscosity is approximately constant along the droplet trajectory. We therefore hypothesize that the distortion from a circular contact area to a distorted elliptical contact area introduces scaling factors, 1/β and 1/(λβ), into the final position and relaxation time, respectively, of the tanh() solution but does not alter the form of the solution.

## Experimental Results and Discussion

### Qualitative Comments on Droplet Motion

**Figure 6:**
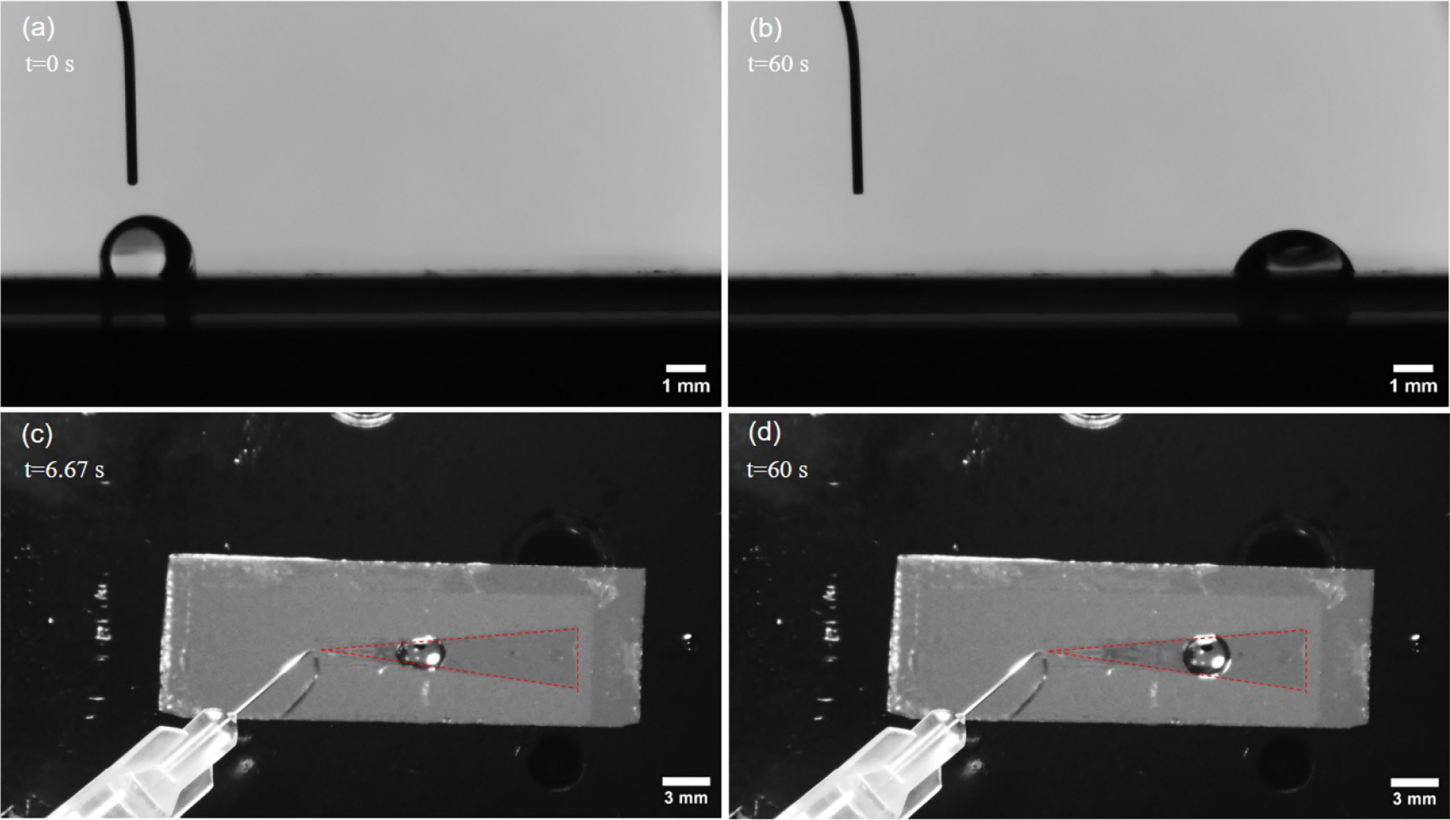
Example side and top views of a droplet at two positions as it traverses along a composite SLIPS wedge from the apex to its equilibrium position (see also Supporting Videos 2 and 3). Side-profile views showing (a) the initial circular arc profile without visible wetting ridges and (b) the final circular arc profile with small wetting ridges. Top views showing (c) a distorted elliptical profile with maximum transverse width indicated and (d) a profile faceting along the edge of the wedge. In parts (c) and (d), the red dashed lines are a guide to the eye showing the lubricant boundaries between the inner wedge region (olive oil) and the surrounding regions (Krytox).

Here, we summarize our observations for the motion of 4 μL droplets on the 11 composite wedge-shaped olive oil/Krytox SLIP surfaces for wedge opening half angles from ξ = 4° to ξ = 14°. Typically, a droplet rapidly moved from the apex fully onto the wedge and self-propelled along the center line of the wedge until coming to rest. The total distance of travel was between 5.6 and 11.2 mm and increased systematically as the wedge opening half angle decreased. We observed that droplets had side-profile-view shapes conforming to circular arcs and initially had no visible wetting ridges at their contact lines (Figure 6a). However, in some cases, small wetting ridges developed either during motion or once the droplets came to rest (Figure 6b). We observed that if a sample had any regions of excess oil in the path of the droplet motion, this oil appeared to be swept into the advancing front edge of the droplet, contributing to a final wetting ridge that was slightly larger on the front edge of the droplet furthest from the apex of the wedge. We note the initial contact angles were around 118° ± 2° and the final contact angles estimated by the intersection between the circular arc profile and the substrate were in the range 66°–76°. This suggests in these samples that the droplet was on excess films of oil^22,47^ and so below the 83.9° contact angle for droplets on thin films of the more wettable olive oil (Supporting Information). At the distance when motion stopped, the difference in estimated contact angle between the two sides of a droplet was 0° ± 2°. Due to the droplet motion across the field of view and its effect on the backlight providing a silhouette of the droplet with a clear outline, the differences were within the accuracy of measurement. However, we were able to identify profiles of droplets and obtain the center position of droplets throughout the full time scale of each experiment.

Observed from the top-view video, each droplet initially elongated along the direction of motion with a larger leading edge compared to that of the trailing edge (Figure 6c). Characterizing this by the ratio of the maximum transverse droplet width to its length, this distortion was droplet dependent but could reach ∼1.6 before relaxing back toward a ratio in the range 1.01–1.51 with the largest of these initial and final values corresponding to the smallest wedge opening half angles. We also observed that despite our attempts to create substrates with stable thin films of oil, repeated experiments on a single substrate appeared to cause depletion of oil. From the side view, once a droplet was fully on a wedge, it appeared to have a contact radius, which for our surfaces and droplet volume gave an average across multiple samples of *r* = 1.6 ± 0.1 mm. An element of faceting along the two wedge sides was observed, and this was most pronounced when the droplet completed its motion (Figure 6d). We are also aware that with a wetting ridge developing as droplets travel along the wedge, the droplet edge will span across the wedge boundary rather than being a sharply defined contact point at the boundary between the inner and outer regions of wettability.

### Position-Time Data for Droplet Motion

**Figure 7:**
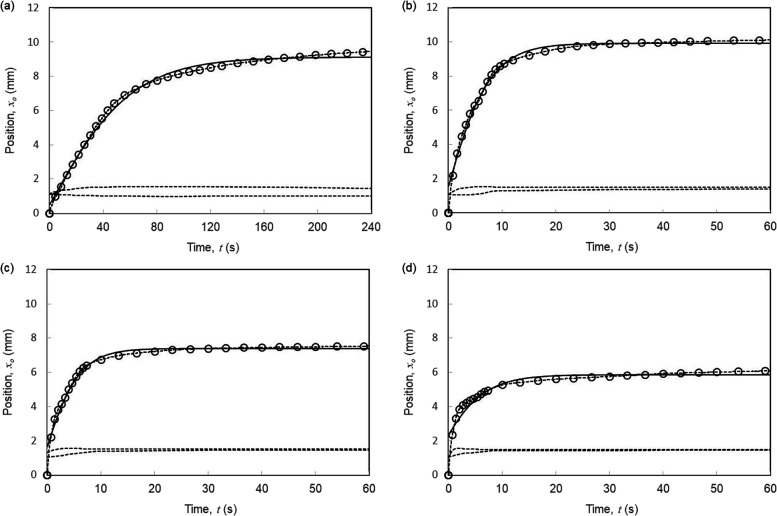
Examples of motion of 4 μL water droplets self-propelling on composite wedge-shaped SLIP surfaces with wedge opening half angles of ξ = 5, 7, 9, and 11° for panels (a), (b), (c), and (d), respectively. In each case, the dotted line is the measured data series of droplet center position as a function of time with the open circle symbols selected representative data points from the series. The solid lines are fits to eq 20, *x*_o_(*t*)=*x*_f_tanh((*t* + *t*_i_)/τ), using three parameters *t*_i_, τ, and *x*_f_, and the horizontal dashed lines are the measured length and the maximum transverse radius of the droplet (from top view images).

Figure 7 shows the evolution with time for the center of a droplet (from side-view images) on composite SLIPS wedges with four different wedge opening half angles: 5, 7, 9, 11°. A full set of figures for the droplet motion on the 11 samples are given in the Supporting Information (Figure S5). The data show the motion of the third droplet on the sample to allow a compromise between droplets sweeping out any local inhomogeneity and depleting lubricant after multiple runs. Because there are at least 3600 experimental data points extracted from each video sequence, the data are plotted on each graph as a dotted line with selected representative data points shown by open symbols. The lower dotted curves in each plot also show the maximum transverse width of the droplet and the droplet length along the wedge measured from the top view. The top views show how the asymmetry in the droplet initially increases and then decreases as the droplet approaches its final position. For each of the 11 data sets, the droplet base radius (assessed from the side profile view data) was well-approximated by a constant for each droplet self-propelled motion sequence.

We found that experimental data could be reasonably fitted by the analytical tanh() solution (eq 20) and these fits are shown by the solid lines through the data points; the fitting parameters *t*_i_, *x*_f_*,* and τ are given in Table 4. Visually, the best fits were found for wedge opening half angles from 4 to 9°, with the tanh() fit struggling to capture the early time data as the wedge opening half angle further increased from 10° to 14°. By restricting the data to later times, more accurate curves for the later time range could be fitted, which is unsurprising as the droplet needs to be fully on the wedge for the theoretically modeled capillary driving forces to occur from both the front and back of a droplet. Moreover, as the droplet travels less distance for larger wedge opening half angles, there is less time for self-centered and dynamic equilibrium to be reached and the small wedge opening half angle approximation becomes less valid. It is clear from Figure 7 that the analytical tanh() solution is surprisingly effective in describing the motion of the center of the droplet over a wide range of wedge opening half angles despite the extent of assumptions used in deriving the theory.

**Table 4:** Fitting and Predicted Parameters

wedge half angle, ξ (°)	initial time, *t*_i_ (s)	final position, *x*_f_ (mm)	time constant, τ (s)	ave. base radius, *r* (mm)	initial contact angle, θ_o_ (°)	final contact angle, θ_i_ (°)	ave. viscosity, <η_s_> (mPa·s)	scaling, *x*_f_/*x*_mf_	scaling, τ/τ_m_
4.0	–11.16	10.88	61.61	1.72	120	66.1	63.7	0.313	4.13
5.0	–3.53	9.12	63.69	1.53	120	69.7	64.2	0.368	6.96
6.0	–1.61	10.58	12.43	1.60	120	72.7	66.3	0.489	1.70
7.0	–1.37	9.92	8.56	1.53	118	75.7	67.9	0.560	1.48
8.0	–0.83	8.35	5.42	1.53	120	73.8	67.8	0.538	1.33
9.0	–1.60	7.40	6.97	1.62	120	75.2	67.1	0.507	2.01
10.0	–1.39	8.14	6.16	1.55	116	74.2	70.3	0.651	2.08
11.0	–3.86	5.86	9.09	1.56	117	74.0	67.2	0.512	3.88
12.0	–3.47	6.79	7.48	1.55	120	69.2	70.4	0.650	4.32
13.0	–4.32	5.99	9.04	1.54	118	72.7	69.5	0.623	5.60
14.0	–1.81	5.53	5.21	1.56	120	72.4	69.4	0.613	3.87

**Figure 8:**
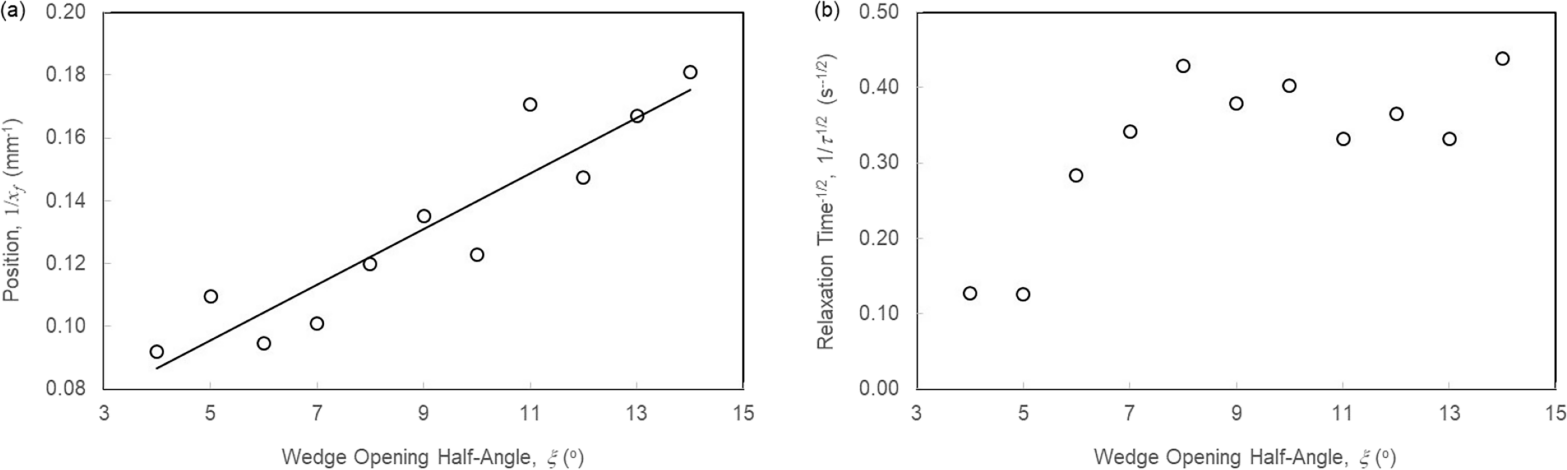
Dependence of the fits for the final position, *x*_f_, and relaxation time, τ, on wedge opening half angles, ξ. Testing of scaling laws from eqs 21 and 22: (a) 1/*x*_*f*_ ∝ ξ and (b) 1/τ^1/2^ ∝ ξ.

According to eq 21, the distance traveled by a droplet, and its final position *x*_f_, should scale inversely with the wedge opening half angle, i.e., *x*_f_ ∝1/ξ. The experimental data in Table 4 plotted in Figure 8a show that this scaling is obeyed in our experiments with droplet volumes of 4 μL for the wedge opening half angles in the range 4°–14°. According to eq 22, the relaxation time, τ, should scale as an inverse square law of the wedge opening half angle, i.e., τ ∝1/ξ,^2^ but Figure 8b suggests the data are inconclusive. If data for wedges with ξ = 9°–14° is excluded, one might argue that the trend in data supports the expected scaling but equally the data for ξ = 8° to 14° could be interpreted as suggesting a saturation to a set of values scattered around 0.38 ± 0.05.

### Expectations from Material Parameters

The circular contact area model should provide order of magnitude expectations for the expected fitting parameters, *x*_f_ and τ, from the substrate materials and wedge design using eqs 14 and 15. For our wedge designs, the contact angles on thin films of olive oil inside the wedge and Krytox outside the wedge were measured to be θ_i_ = 66–76° and θ_o_ = 118 ± 2°, respectively (Table 4). The interfacial tensions in Table 2 suggest that a droplet in contact with a composite substrate of olive oil and Krytox will be double-cloaked with an inner film of olive oil and an outer film of Krytox and so have an effective drop-vapor surface tension of γ_DV_= γ_WOO_+ γ_OOK_ + γ_KV_ = 51.3 mN/m. To estimate the average lubricant meniscus viscosity < η_s_ > for each droplet experiment on a wedge, we use the ratio of circular arc segments inside the wedge to the overall circular arc, i.e.,
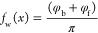
23The average viscosity for a droplet traveling a distance, *d*, along a wedge was then assumed to be
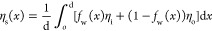
24The viscosities of water, olive oil, and Krytox used in eq 24 were taken from the literature as η_w_ = 1.0 mPa·s, η_i_ = 60.0 mPa·s, and η_o_ = 75.0 mPa·s, respectively.^48^ This gave average viscosities in the narrow range 64–70 mPa·s, which means the order of magnitude estimates are insensitive to whether eq 24 is valid or not. Finally, a value of α = 27 was used in eq 6 for all data sets for the viscous dissipation.^15,61^ Based on these parameters, eq 14 gives a plausible order of magnitude estimate for the measured *x*_f_ but overpredicts the distance traveled by factors ranging from 3.2 to 1.6 as the wedge opening half angle increases (with an average of 2.0 across the 11 data sets). Similarly, eq 15 gives a plausible order of magnitude estimate for the measured τ but overpredicts the relaxation time by factors ranging from 1.3 to 6.6 (with an average of 2.0 across the 11 data sets).

### Possible Wider Applicability of the Tanh() Solution

**Figure 9:**
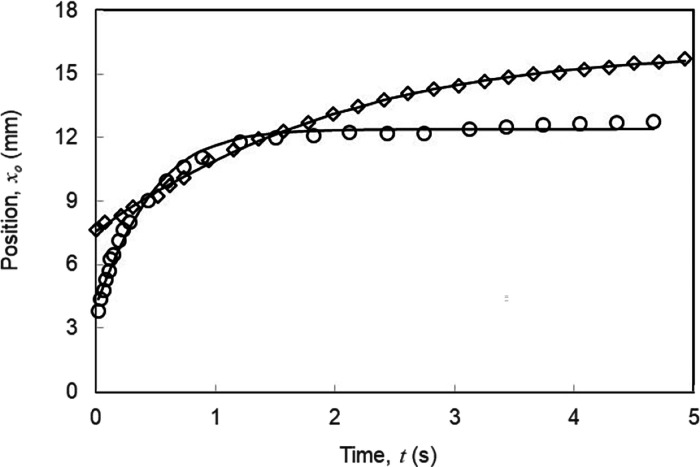
Motion of ca. 5 μL water droplets self-propelling on copper foil wedge-shaped pieces treated to create superhydrophobic CuO-PDMS oil-infused SLIP surfaces with opening half angles of ξ = 3° (diamond symbols) and ξ = 6° (circle symbols). The data is from Zheng et al.^46^, and the solid lines are fits to eq 20, *x*_o_(*t*)=*x*_f_tanh((*t* + *t*_i_)/τ), using three parameters *t*_i_, τ, and *x*_f_.

One of the most surprising aspects of the theoretical analysis is the extent to which a simple tanh() curve appears to describe each data set despite the extent of assumptions used. This appears to be linked to the general observation that the front and back edges of a droplet will be portions of smooth arcs (approximated by ellipses), which intersect the boundaries between more and less wettable regions of the wedge. Identifying these points and keeping the wedge opening half angle small tend to result in a capillary force proportional to ξ(1 – β^2^ξ^2^*x*_o_^2^/2*r*^2^). Provided the dominant forces are viscous dissipation and this is the form of capillary force, the solution will be tanh(), which physically corresponds to an initial constant speed, which then exponentially approaches zero (see eq 17). To test whether the tanh() solution (eq 20) might have wider applicability than our experiments, we also fitted the literature data of Zheng et al.^46^ on water droplet motion on a wedge-shaped superhydrophobic copper surface combined with a poly(dimethylsiloxane) (PDMS) oil layer on it (Figure 9). We were able to obtain excellent fits, which suggests that the tanh() model may have wider applicability to wedge-shaped gradient wettability systems, albeit with modifications for non-SLIP surfaces where contact line pinning may be significant. It would be interesting to test whether literature data for droplet motion on various wedge geometries not involving SLIPS and for liquids filling wedge-shaped regions might be described empirically by eq 20.

## Conclusions

In this work, we have developed a dewetting approach to creating patterned substrates with materials, Teflon AF1600 and a superhydrophobic nanoparticle coating (Glaco), capable of stabilizing two different infused-liquids, which act as lubricants for a composite slippery liquid-infused porous surface (SLIPS). We have shown that it is possible to design and implement patterned composite SLIPS, which allow control of the wettability of the surfaces as predicted by the liquid Young’s law. We have argued that droplets on composite SLIPS can have cloaking effects for the droplet–vapor interface analogous to single- and double-interface core–shell compounds. We have shown that droplets on a composite two-lubricant oil SLIPS allow self-propelled droplet transport toward the spatial region dominated by the oil with higher wettability with little pinning or resistance to motion for the droplet. On wedge-shaped patterns of lubricants with the inner lubricant more wettable, droplets self-propel from the apex toward the broader end of the wedge driven by the wettability gradient. For this surface pattern, we have derived a simple model predicting self-propelled droplet motion obeying a position–time curve described by a tanh() law. Experimental data is well-fitted by the law with the correct order of magnitudes predicted for the final position and the relaxation time in the motion from the wedge design, lubricant, and droplet parameters. This study also highlights the complexity of design considerations needed to ensure that a composite SLIP surface is absolutely stable under different droplet liquids as well as in air.

## Supporting Information Available

Scanning electron microscope (SEM) images of a binary GLACO-Teflon AF1600 surface, side profile images of droplets of different liquids on various SLIP surfaces, images showing preferential wetting by a water droplet of olive oil-based SLIPS over Krytox-based SLIPS, derivation of capillary forces on a wedge-shaped wettability pattern, and position-time data for droplet motion on wedge–shaped SLIPS patterns (PDF)
Video 1: Side-view deposition of a 4 μL DI water droplet on the boundary between olive oil and Krytox regions of a composite SLIPS supporting Figure 4 (MP4)
Video 2: Example droplet motion on the wedge-shaped olive oil region within a Krytox background viewed from the side corresponding to images in Figure 6 (MP4)
Video 3: Example droplet motion on the wedge-shaped olive oil region within a Krytox background viewed from the top corresponding to images in Figure 6 (MP4)
Video 4: Axonometric view of a 4 μL DI water droplet motion from the apex (tip) of a wedge-shaped olive oil region within a Krytox background toward the wider end of the wedge (without annotation so that boundaries between the two oils can be seen) (MP4)
