# Supplementary material for: Droplet Self-Propulsion on Slippery Liquid-Infused Surfaces with Dual-Lubricant Wedge-Shaped Wettability Patterns
Source: Langmuir. 2023 Oct 24;39(44):15676–89. doi: 10.1021/acs.langmuir.3c02205 (PMC10634355; doi:10.1021/acs.langmuir.3c02205)
Supplement: Supplementary file 1 — la3c02205_si_001.pdf [file la3c02205_si_001.pdf]

Supplementary Information

Droplet Self-Propulsion on Slippery Liquid-Infused Surfaces with Dual Lubricant Wedge-Shaped Wettability Patterns

Michele Pelizzari[1], Glen McHale*[1], Steven Armstrong[1], Hongyu Zhao[1], Rodrigo Ledesma-Aguilar[1], Gary G. Wells[1], and Halim Kusumaatmaja[2]

[1]Institute for Multiscale Thermofluids, School of Engineering, The University of Edinburgh, Edinburgh EH9 3FB, UK. [2]Department of Physics, Durham University, Durham DH1 3LE, UK.

*Email: glen.mchale@ed.ac.uk

TABLE OF CONTENTS

1. Dewetting of Glaco from Teflon AF1600 S2
2. Contact Angles for Droplets on Different Surfaces S3
3. Drop Motion to a SLIPS Region of Lower Wettability S3
4. Derivation of Capillary Forces on a Wedge-Shaped Wettability Region S4
5. Droplet Motion on the Eleven Wedge-Shaped Wettability Patterned SLIP Surfaces S7
6. Description of Supplementary Videos S7

1. Dewetting of Glaco from Teflon AF1600

The dewetting of the Glaco superhydrophobic solution from Teflon AF1600 coated areas of substrates was confirmed using Scanning Electron Microscopy (SEM) images of substrates after lithographic patterning. Figure S1 shows a surface patterned with one side coated with Teflon AF1600 and one side with Glaco (i.e. a binary surface).

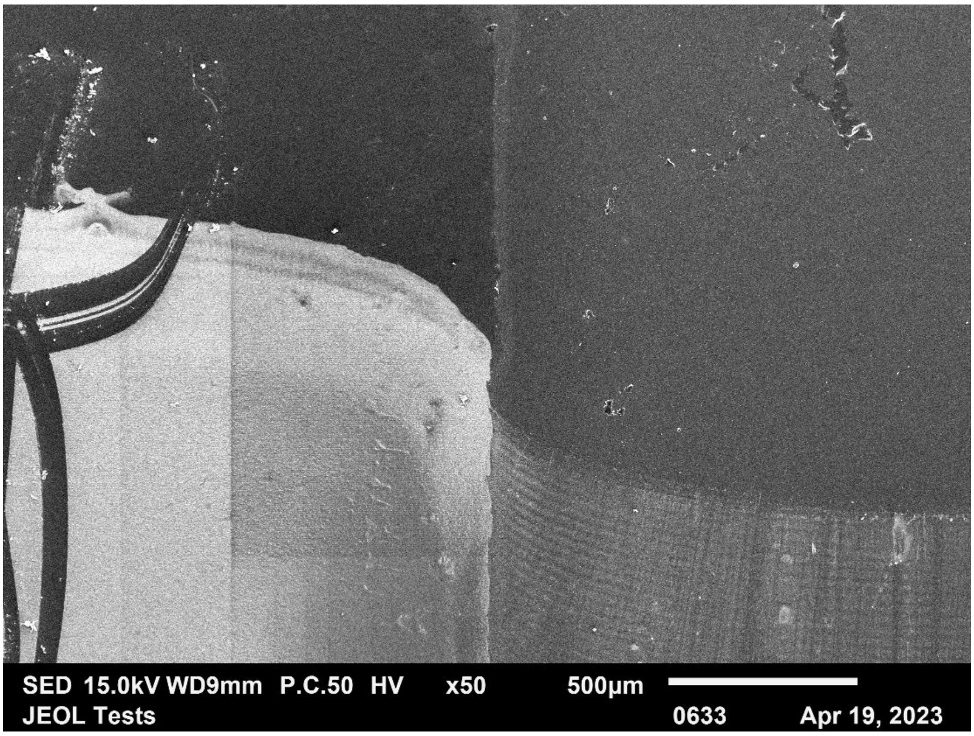

Figure S1. Scanning electron microscope (SEM) images of a binary GLACO-Teflon AF1600 surface. The right half of the sample is first coated with Teflon. Subsequently, the all sample is dip coated in Glaco five times (U_{in}=1.00 mm/s, U_w=1.00 mm/s). After the five coatings, GLACO develops in a thick coating layer on the left side of the sample (white region), where no Teflon is present. On the right side of the sample, the Teflon prevents the silica nanoparticles attaching to the surface, with only a small amount depositing which does not change the superficial chemistry of the region.

2. Contact Angles for Droplets on Different Surfaces

Example side profile view images of droplets on various surfaces are given in Fig. S2.

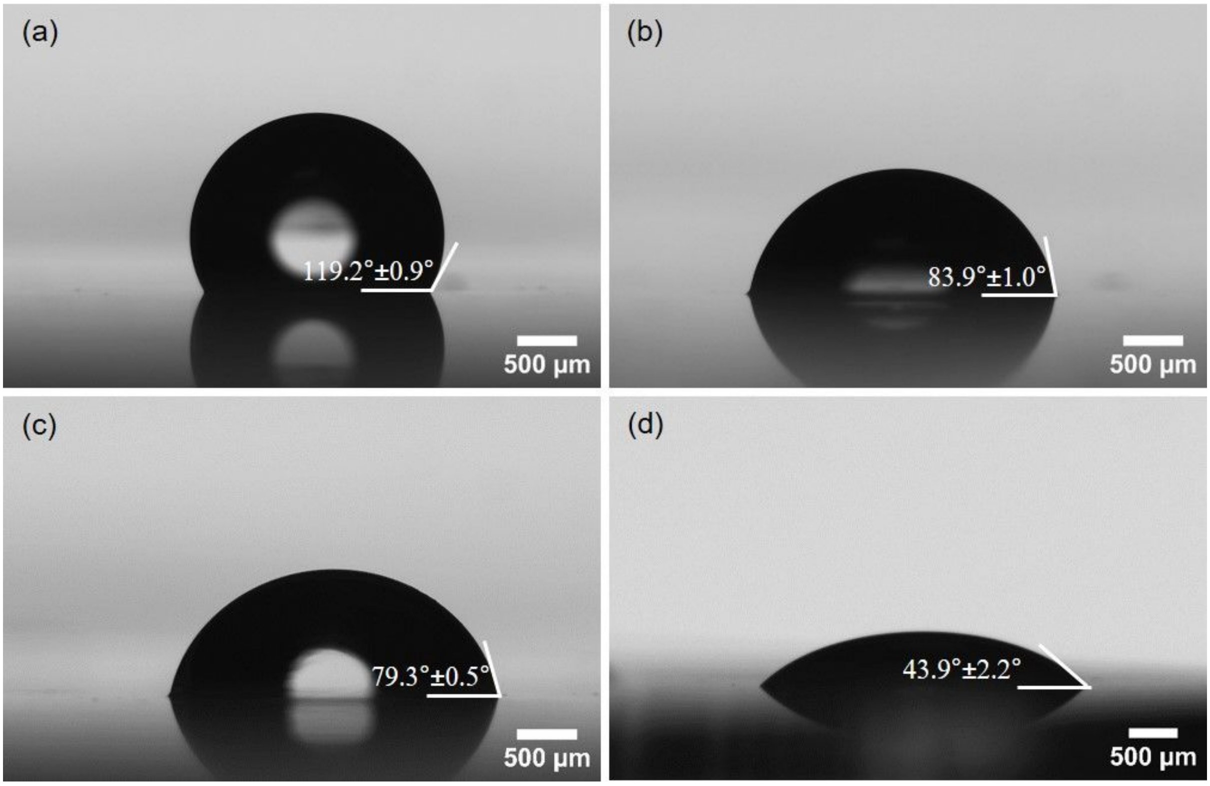

Figure S2. Images of 4 µl droplets on different types of surfaces. (a) DI water on Krytox Teflon AF1600-based SLIPS, (b) DI water on Olive oil Glaco-based SLIPS, (c) Olive oil droplet on a Krytox Teflon AF1600-based SLIPS, (d) Krytox on Olive oil Glaco-based SLIPS.

3. Drop Motion to a SLIPS Region of Lower Wettability

Figure S3 shows a sequence of side profile images for a droplet of water deposited on the boundary between an Olive oil-based SLIPS region and a Krytox-based SLIPS region. The droplet rapidly moves across so it rests entirely on the Olive oil-based SLIPS which has the lower value of (liquid) Young's law contact angle.

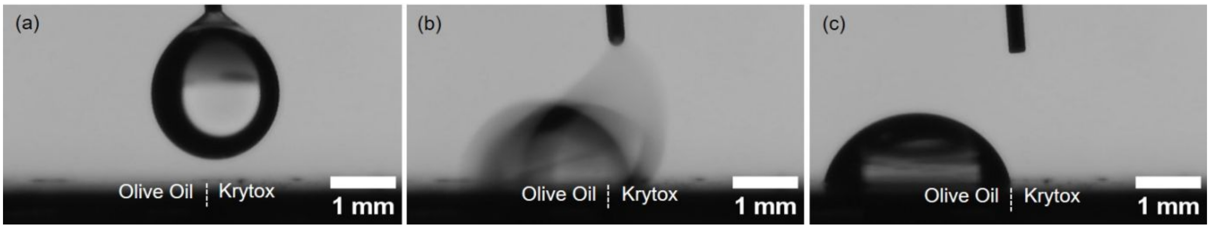

Figure S3. Side view of a 4 µl DI water droplet deposited on the boundary region between Olive oil and Krytox on a OOG-KT Composite SLIPS. (a) The droplet is still attached to the needle. (b) The droplet touches the surface and detaches from the needle with an immediate movement. (c) The droplet sits on the Olive oil region due to the lesser contact angle.

4. Derivation of Capillary Forces on a Wedge-Shaped Wettability Region

Circular Geometry and Coordinate System. The coordinate system is presented in Figure S4. We consider an *x*-*y* plane reference system, with a wedge pattern defined by the half-angle at the wedge ξ. We consider the tip of the wedge being at the origin of the reference system, with the wedge symmetrical to the x-axis. The droplet footprint is a circumference defined by a constant radius *r* and by the centre x_0. We assume that the droplet centre x_0 moves alongside the *x*-axis, with the droplet always remaining on the wedge main axis. The intersection points between the circumference and the wedge are defined by the angles at the centre φ_b and φ_f.

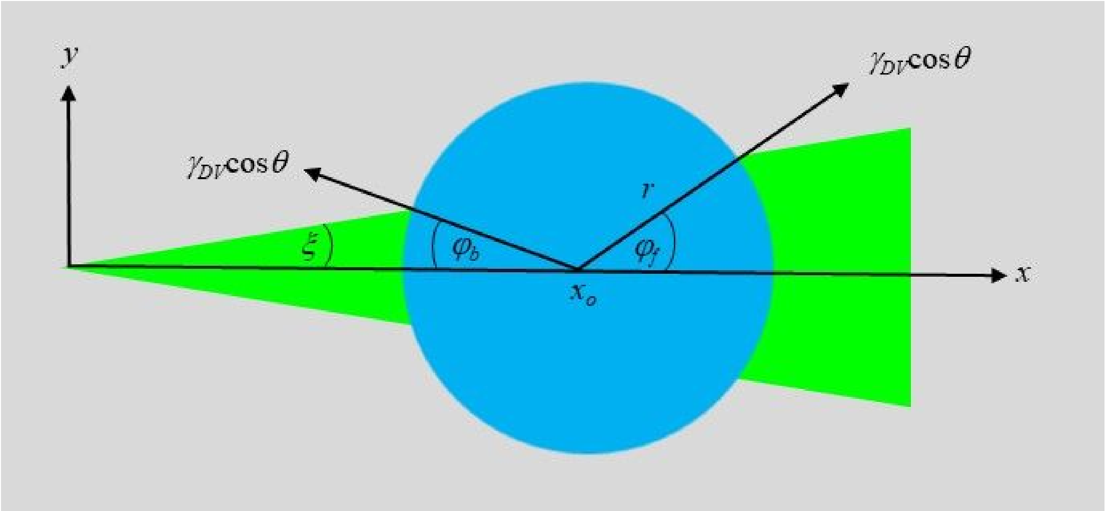

Figure S4. Schematic representation of the wedge geometry considering a circular droplet footprint. x_0 is the geometrical centre of the droplet. ξ is the half-angle at the wedge, φ_b and φ_f are the angles on the *x*-*y* plane defined by the intersections between the droplet footprint and the wedge geometry; the subscripts *b* and *f* refer to the back and front of the droplet, under the assumption that the droplet moves in the positive direction on the *x*-axis. $\gamma_{DV}\cos\theta$ is the projection of the capillary force on the *x*-*y* plane.

Capillary Forces. The net force from the droplet on the wedge area is given by integrating from 0 to the wedge-circle intersection,

$$F_i = 2\left[\int_0^{\varphi_f} \gamma_{DV}\cos\theta_i \cos\varphi r d\varphi - \int_0^{\varphi_b} \gamma_{DV}\cos\theta_i \cos\varphi r d\varphi\right] \quad \text{(S1)}$$

where θ_i is the contact angle on the inside of the wedge. This gives

$$F_i = 2r\gamma_{DV}\cos\theta_i(\sin\varphi_f - \sin\varphi_b) \quad \text{(S2)}$$

Similarly, for the forces outside the wedge integrate from the wedge-circle intersection to 90°,

$$F_o = 2r\gamma_{DV}\cos\theta_o(\sin\varphi_b - \sin\varphi_f) \quad \text{(S3)}$$

where θ_o is the contact angle on the outside of the wedge The total force on the droplet is then,

$$F_{Net} = 2r\gamma_{DV}(\cos\theta_o - \cos\theta_i)(\sin\varphi_b - \sin\varphi_f) \quad \text{(S4)}$$

Evaluation of Wedge Geometry Factor. The geometry factor is encoded within φ_b and φ_f and these depend on the wedge half-angle ξ, the drop position x_o and droplet base radius r. Equations for edge of wedge and for the droplet base perimeter positions are,

$$y = x\tan\xi \tag{S5}$$

$$(x - x_o)^2 + y^2 = r^2 \tag{S6}$$

and the intersection defining the angles φ_f (and φ_b) is given by,

$$\sin\varphi_f = \frac{y}{r} \tag{S7}$$

Using eq S5 and eq S7 gives,

$$\sin\varphi_f = \frac{x\tan\xi}{r} \tag{S8}$$

and using eq S5 and eq s6 allows the two roots of x to be found,

$$(x - x_o)^2 + x^2\tan^2\xi = r^2 \tag{S9}$$

Expanding and grouping terms,

$$x^2(1 + \tan^2\xi) - 2xx_o + (x_o^2 - r^2) = 0 \tag{S10}$$

Solutions of the quadratic are,

$$x = \frac{x_o \pm [x_o^2 - (1 + \tan^2\xi)(x_o^2 - r^2)]^{1/2}}{(1 + \tan^2\xi)} \tag{S11}$$

i.e.

$$x = \frac{x_o \pm [r^2 - (x_o^2 - r^2)\tan^2\xi]^{1/2}}{(1 + \tan^2\xi)} \tag{S12}$$

Equation S8 then gives,

$$\sin\varphi_{f,b} = \left\{\frac{x_o \pm [r^2 - (x_o^2 - r^2)\tan^2\xi]^{1/2}}{r(1 + \tan^2\xi)}\right\}\tan\xi \tag{S13}$$

where the ± roots correspond to the front and back of the droplet. The total force on the droplet is then given by eq S14,

$$F_{Net} = 4r\gamma_{DV}(\cos\theta_i - \cos\theta_o)\tan\xi\left\{\frac{[r^2 - (x_o^2 - r^2)\tan^2\xi]^{1/2}}{r(1 + \tan^2\xi)}\right\} \tag{S14}$$

i.e.

$$F_{Net} = 4r\gamma_{DV}(\cos\theta_i - \cos\theta_o)\tan\xi\left\{\frac{\left[1 + (1 - x_o^2/r^2)\tan^2\xi\right]^{1/2}}{(1 + \tan^2\xi)}\right\} \quad \text{(S15)}$$

The approximate case is given by expanding in small wedge angle ξ,

$$F_{Net} \approx 4r\gamma_{DV}(\cos\theta_i - \cos\theta_o)\xi\left(1 - \frac{1}{2}\left(1 + \frac{x_o^2}{r^2}\right)\xi^2 + \ldots\right) \quad \text{(S16)}$$

This final equation is of the form for the capillary force that gives a tanh() solution for the position of the droplet with time when self-propelling along the wedge.

Elliptical Geometry Modification for Small Wedge Half-Angles. If the droplet is elongated along the direction of the wedge, we consider an elliptical approximation to both the front and back perimeter shapes. Using the ratio $\beta=r/b$ where $2r$ is the major axis width and $2b$ is the minor axis width, the formula for an ellipse centred along the wedge is,

$$(x - x_o)^2 + \beta^2 y^2 = r^2 \quad \text{(S17)}$$

When $\beta=1$, eq S17 reduces to eq S6 in the calculation for a circle. Assuming a small wedge half-angle we retain eq S7 and the ellipse equivalent expression to eq. S13 is then,

$$\sin\varphi_{f,b} = \left\{\frac{x_o \pm \left[r^2 - (x_o^2 - r^2)\beta_{f,b}^2\tan^2\xi\right]^{1/2}}{r(1 + \beta_{f,b}^2\tan^2\xi)}\right\}\tan\xi \quad \text{(S18)}$$

Assuming the effective radius is approximately the same at the front and back we write,

$$\sin\varphi_f - \sin\varphi_b = \left\{\frac{\left[(x_o^2 - r^2)\beta_f^2\tan^2\xi\right]^{1/2}}{r(1 + \beta_f^2\tan^2\xi)} + \frac{\left[(x_o^2 - r^2)\beta_b^2\tan^2\xi\right]^{1/2}}{r(1 + \beta_b^2\tan^2\xi)}\right\}\tan\xi \quad \text{(S19)}$$

which for small wedge angles is,

$$\sin\varphi_f - \sin\varphi_b = \xi\left(1 - \frac{1}{2}\left(1 + \frac{x_o^2}{r^2}\right)\left(\frac{\beta_f^2 + \beta_b^2}{2}\right)\xi^2 + \ldots\right) \quad \text{(S20)}$$

The approximate net force is then assumed to be given by,

$$F_{Net} \approx 4r_{eq}\gamma_{DV}(\cos\theta_i - \cos\theta_o)\xi\left(1 - \frac{1}{2}\left(1 + \frac{x_o^2}{r^2}\right)\beta^2\xi^2 + \ldots\right) \quad \text{(S21)}$$

where

$$\beta^2 = \frac{\beta_f^2 + \beta_b^2}{2} \quad \text{(S22)}$$

and $2\pi r_{eq}$ is assumed to be an average effective perimeter length for an ellipse. The assumptions in this elliptical approximation are strong, but suggest a tanh() solution for position with time modified by a β factor in eq S21 resulting in a scaling of both the final position, x_f, and the relaxation time, τ.

5. Droplet Motion on the Eleven Wedge-Shaped Wettability Patterned SLIP Surfaces

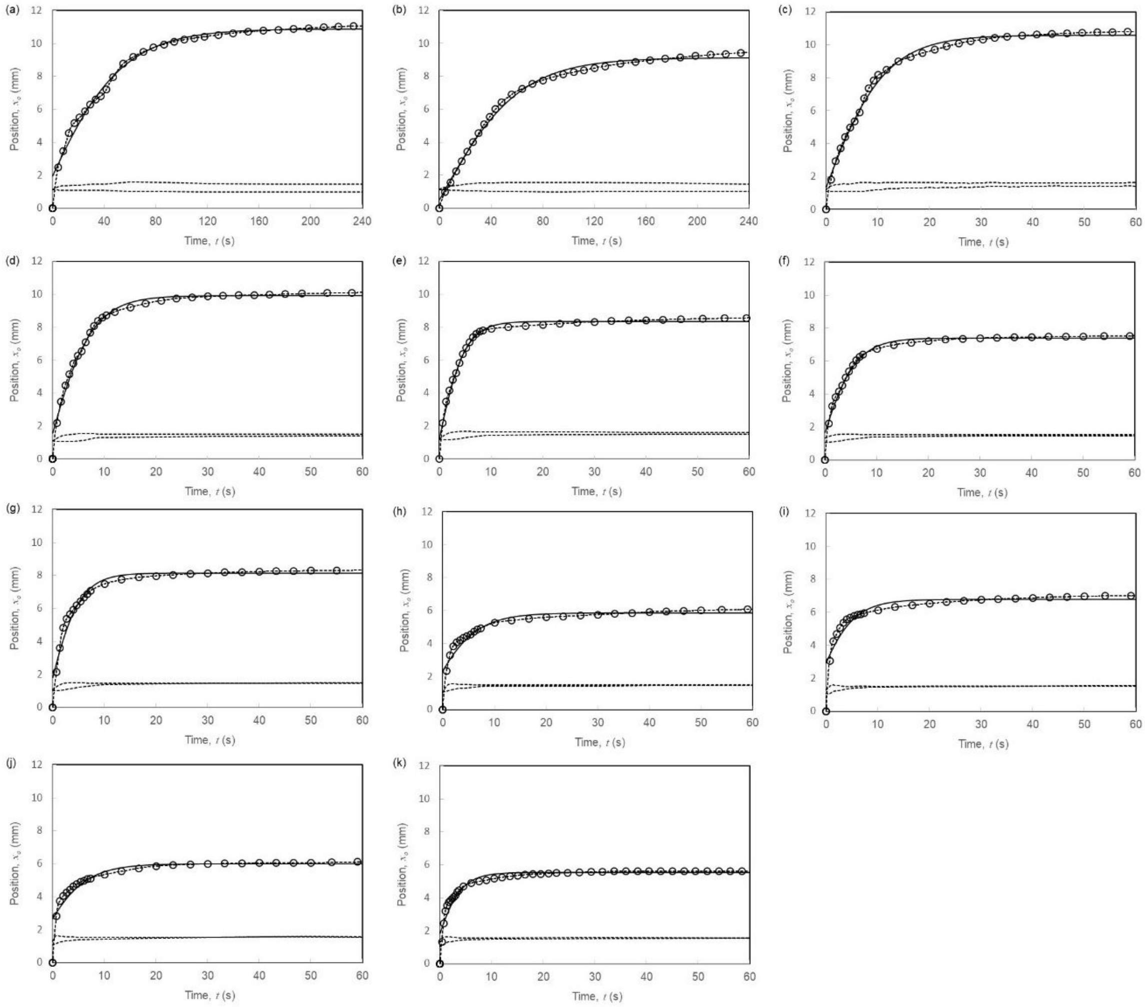

Figure S5. Motion of 4 μl water droplets self-propelling on composite wedge-shaped SLIP surfaces with wedge opening half-angles of $\xi=4^o$, 5°, 6°, 7°, 8°, 9°, 10°, 11°, 12°, 13° and 14° for panels (a)-(k), respectively. In each case, the dotted line is the measured data series of droplet centre position as a function of time with the open circle symbols selected dated points from the series. The solid lines are fits to eq 20, $x_o(t)=x_f\tanh(t+t_i)/\tau)$, using three parameters t_i, τ and x_f, and the horizontal dashed lines are the measured length and the maximum transverse radius of the droplet (from top view images).

6. Description of Supplementary Videos

Video 1. The video shows the side view of a 4 μl DI water droplet deposited over an Olive oil (left) and Krytox (right) region. The droplet immediately moves onto the Olive oil region due to the preferential wettability of the droplet on Olive oil compared to Krytox.

Video 2 and Video 3. The videos show the side view and the top view of a 4 μl DI water droplet deposited over the tip (apex) of a wedge-shaped Olive oil pattern in a Krytox background. The droplet self-propels towards the wider end of the wedge. When the droplet no longer has any contact with the

Krytox, the motion stops since the droplet sits completely on an Olive oil region and there is no wettability difference. Annotation has been added to identify the wedge-shaped region of Olive oil.

Video 4. The video shows a view from the top and to the side of a 4 µl DI water droplet deposited over the tip (apex) of a wedge-shaped Olive oil pattern in a Krytox background and self-propelling until it rests entirely on the more wettable Olive oil region towards the wider end of the wedge. This video has not had any annotation added and so allows the contrast between the two oils to be seen directly without being obscured by annotation.
